# Examination of Caregiver Logistics in a National Sample of Family Caregivers of Older Veterans

**DOI:** 10.1093/geroni/igaf122.910

**Published:** 2025-12-31

**Authors:** Victoria Ngo, Steven Shirk, Elizabeth Marfeo, Elizabeth Chamberlin, Bret Hicken, Maria Venegas, Leah Christensen, Lauren Moo

**Affiliations:** VA Bedford Health Care System, Bedford, Massachusetts, United States; VA Bedford Health Care System, Bedford, Massachusetts, United States; Tufts University, Medford, Massachusetts, United States; VA Bedford Health Care System, Bedford, Massachusetts, United States; VHA Office of Rural Health, Salt Lake City, Utah, United States; Department of Veterans Affairs, Bedford, Massachusetts, United States; Veterans Health Administration, Washington, District of Columbia, United States; VA Bedford Healthcare System, Bedford, Massachusetts, United States

## Abstract

Family caregivers of older Veterans face challenges in coordinating visit-associated logistics. Based on a national survey we developed, we were able to learn more about some of these challenges. In this talk, we share results as we examined caregiver logistics of attending a medical visit. Descriptive statistics including means, standard errors, and percentages, were used to summarize the sociodemographic characteristics of caregiver respondents. Associations between caregiver and care recipient characteristics and caregiver burden were examined in bivariate analyses using chi-square tests of independence for categorical variables and t-tests for continuous variables. Relationships between variables related to ADL and IADL and visit-associated logistics were examined using t-tests.

